# Discovery of Novel Antiangiogenic Marine Natural Product Scaffolds

**DOI:** 10.3390/md14030057

**Published:** 2016-03-11

**Authors:** Hassan Y. Ebrahim, Khalid A. El Sayed

**Affiliations:** mohamehy@warhawks.ulm.edu

**Keywords:** marine natural products (MNPs), angiogenesis, kinase profiling, molecular modeling

## Abstract

Marine natural products (MNPs) are recognized for their structural complexity, diversity, and novelty. The vast majority of MNPs are pharmacologically relevant through their ability to modulate macromolecular targets underlying human diseases. Angiogenesis is a fundamental process in cancer progression and metastasis. Targeting angiogenesis through selective modulation of linked protein kinases is a valid strategy to discover novel effective tumor growth and metastasis inhibitors. An in-house marine natural products mini-library, which comprises diverse MNP entities, was submitted to the Lilly’s Open Innovation Drug Discovery platform. Accepted structures were subjected to *in vitro* screening to discover mechanistically novel angiogenesis inhibitors. Active hits were subjected to additional angiogenesis-targeted kinase profiling. Some natural and semisynthetic MNPs, including multiple members of the macrolide latrunculins, the macrocyclic oxaquinolizidine alkaloid araguspongine C, and the sesquiterpene quinone puupehenone, showed promising results in primary and secondary angiogenesis screening modules. These hits inhibited vascular endothelial growth factor (VEGF)-mediated endothelial tube-like formation, with minimal cytotoxicity at relevant doses. Secondary kinase profiling identified six target protein kinases, all involved in angiogenesis signaling pathways. Molecular modeling and docking experiments aided the understanding of molecular binding interactions, identification of pharmacophoric epitopes, and deriving structure-activity relationships of active hits. Marine natural products are prolific resources for the discovery of chemically and mechanistically unique selective antiangiogenic scaffolds.

## 1. Introduction

Marine natural products (MNPs) are characterized by their structural novelty, complexity, chirality and diversity. The vast majority are biologically relevant, capable of interacting with biological macromolecules, with high specificity and potency. For many decades, researchers used marine metabolites as chemical probes to interrogate the complex biological systems and/or utilize them as candidates to modulate pharmacologically relevant pathways underlying different diseases [1]. To date, seven marine-derived products have been approved as drugs by the United States Food and Drug Administration (FDA) and/or European Medicines Agency (EMA) to treat various diseases, indicating the remarkable and serious contribution of MNPs to the drug discovery arena [2].

Marine macrolides constitute a prominent class of distinct natural products characterized by a highly oxygenated polyene backbone containing a macrocyclic lactone as a conformational constraint [3]. Latrunculins are marine-derived macrolides produced by sponges of the genus *Latrunculia* (later changed to *Negombata*), whence the name was derived. Latrunculins possess the common scaffold of a 16- or 14-membered macrolactone ring fused with a tetrahydropyran (THP) ring, to which a rare thiazolidinon-2-yl (TZD) moiety is attached. Since their discovery as microfilament disruptors in 1983 by Spector *et al.* [4], various biological activities have been documented for latrunculins, including antiproliferative [5], cytotoxic [6], antimicrobial [7], and anti-invasive [8]. Although biological effects of latrunculins and their semisynthetic analogs were mostly attributed to their actin-binding capabilities, we previously hypothesized that additional macromolecular target(s) are important for their anticancer activities [8,9].

The shikimate-derived sesquiterpene quinones represent a unique class of marine metabolites that garnered a great deal of attention for their versatile biological activities [10]. Puupehenone is a marine-derived sesquiterpene isolated from sponges of the orders Verongida and Dictyoceratida. Structurally, puupehenone has a drimane terpenoid skeleton to which a unique quinone-methide moiety is fused. Puupehenone was reported to be cytotoxic against multiple cancer cell lines, including P-388, A-549, HCT-8, and MCF-7 [11]. The ability of puupehenone to inhibit the endothelial cell differentiation *in vitro* with minimal cytotoxicity, in addition to *in vivo* angiogenesis inhibition of the chick chorioallantoic membrane, was also reported [12].

Araguspongines are *bis*-1-oxaquinolizidine marine-derived alkaloids commonly found in marine sponges of the genus *Xestospongia*. They selectively inhibit the inositol 1,4,5-trisphosphate (IP3) receptor, a calcium channel mainly located at the endoplasmic reticulum [13]. Araguspongine C comprises the common dimeric 1-oxaquinolizidine skeleton, with two hydroxyl groups attached at the junction of the linkers connecting oxaquinolizidine rings. Recently, araguspongine C was identified by our group to induce autophagic death in breast cancer cells through the suppression of c-Met and HER2 receptor tyrosine kinases signaling [14].

Angiogenesis (recruitment of new blood vessels from existing vasculature) is a fundamental feature of many physiological processes, including reproduction, embryonic development, wound healing, and tissue regeneration [15]. Angiogenesis plays a crucial role in several normal physiological processes, but it also mediates pathogenesis of many diseases, including cancer progression and metastasis. In the past few decades, many angiogenesis inhibitors have been created to reduce many tumors’ progression and invasiveness. However, clinical studies showed that resistance development and toxicity are the main challenges facing the development of ideal angiogenesis inhibitors [16,17]. Therefore, continued research to discover novel antiangiogenic scaffolds is urgently needed to overcome the limitations of the current angiogenesis inhibitors.

Protein kinases regulate many cellular processes, including differentiation, proliferation, motility, survival, apoptosis and metabolism. They phosphorylate their target proteins in a specific and efficient manner [18]. Phosphorylation induces a conformational change in the effector proteins and thus modulates their biological activities. A network of cross-talking protein kinases tightly regulates angiogenesis cascades. However, dysregulation of protein kinase signaling pathways frequently result in pathological angiogenesis [19].

The Janus kinases (JAKs) are non-receptor tyrosine kinases, including four members: JAKs1-3 and TYK2. JAKs, which are bounded to intracellular domains of cell surface receptors, are activated upon binding of interferons, interleukins and growth factors to their cognate receptors. In particular, JAK2 represents a critical component of the signaling pathway implicated in cell growth, proliferation, differentiation, survival and apoptosis. These effects are mostly mediated by its downstream effectors’ signal transducers and activators of transcription (STATs) [20,21]. Furthermore, studies demonstrated the strong link between hyperactivated JAK2/STAT axis and vascular endothelial growth factor (VEGF) overexpression [22].

The receptor tyrosine kinase FLT3 (Fms-like tyrosine kinase 3) is a proto-oncogene cytokine receptor belonging to the class III receptor tyrosine kinase (RTKs) family that plays a key role in hematopoiesis [23]. The change from inactive-to-active confirmation of the kinase domain is triggered upon ligand (FL) binding to extracellular module, subsequently promoting receptor homodimerization and transphosphorylation. Phosphorylation of tyrosine residues in the *C*-terminal tail creates recruitment sites for downstream effectors, including STAT5 [24], rat sarcoma viral oncogene homolog (RAS) [25], and phosphatidylinositol-4,5-biphopshate 3-kinase (PI3K) [26], which ultimately promote cell growth, proliferation and survival. The expression of FLT3 is well documented in many tissues, especially in bone marrow and endothelial cells, which is consistent with its important implications in hematopoiesis and angiogenesis [27].

The Eph receptors (initially cloned from erythropoietin-producing hepatocellular carcinoma) are the largest subfamily of RTKs, activated by their cell membrane-anchored ligands of the ephrin family (Eph receptor interacting protein) [28]. Eph receptors regulate multiple phenotypes, including cell shape, migration and adhesion [29]. In particular, EphB4 receptor expressed on tumor cells can interact with ephrinB2 of the endothelium, thereby promoting angiogenesis and tumor growth [30].

The Abelson murine leukemia viral oncogene homolog 1 (ABL1) is a non-receptor tyrosine kinase that has a critical role in various biological processes, including cell motility, morphogenesis, apoptosis and angiogenesis [31]. ABL1 is stimulated by a variety of upstream stimuli, including growth factors, cell-cell adhesion and DNA damage [32]. Pathologically, human ABL1 is involved in chromosomal abnormalities of hematological cancers. For instance, bcr-ABL fusion protein is the hallmark product of a genetic aberration that leads to constitutively active ABL1 kinase in chronic myeloid leukemia (CML) patients. Furthermore, activation of c-ABL1 by the proangiogenic factor, basic fibroblast growth factor (bFGF), promoted endothelial cell proliferation, survival in response to serum-deprivation, motility, tube formation, angiogenesis, and tumor growth of the triple negative MDA-MB-231 breast cancer cells in a xenograft model [33].

Tyrosine kinases with immunoglobulin-like and epidermal growth factor (EGF)-like domains (TIE1 and TIE2) are endothelial-specific RTKs regulating blood vessels branching and maintaining endothelial homeostasis [34]. TIE2 receptor is the second major endothelial RTK after vascular endothelial growth factor receptors (VEGFRs) essential for maintaining endothelial homeostasis and promoting angiogenesis [35]. The protein growth factor angiopoietins (Ang1–4) modulate TIE receptor activities. TIE2 receptor activation results in recruitment of growth factor receptor-bound protein 2 (Grb2) [36] and PI3K [37], thus promoting endothelial cell motility and survival. In addition, Ang1/TIE2 signaling pathway plays an important role in stabilizing the immature endothelial cell network, attracting pericytes and maintaining vessels integrity [38].

Glycogen synthase kinase-3 beta (GSK-3β) is a multifunctional serine/threonine kinase that plays a key role in the regulation of numerous cellular processes such as cell cycle, inflammation and cell proliferation [39]. GSK-3β regulates endothelial cell migration and survival through induction of proangiogenic factors, vascular endothelial growth factor and fibroblast growth factors [40].

The polo-like kinases (PLKs) are a family of serine/threonine kinases described first in a screen for mutants affecting spindle pole behavior in *Drosophila melanogaster* (polo), from which the name is derived [41]. It comprises four members, PLKs1–4. Among them, PLK1 is the best characterized. PLK1 plays key critical roles in various stages of mitosis, such as bipolar spindle formation, trigger for G2/M transition, chromosome segregation and cytokinesis [42]. Recently, Kushner and co-workers reported the correlation between PLK1 blockade and disruption of endothelial cell migration via suppression of microtubule nucleation and centrosome scattering [43].

This study demonstrates an integrated collaboration between academia and industry for effective biological screening and drug discovery based on natural products. Eli Lilly offered the academic programs access to novel phenotypic assays for new entity discovery and lead generation covering wide areas of human diseases. In this regard, members of an in-house marine natural product-based mini-library, which comprises 71 compounds with diverse entities (macrolides, sesquiterpenes, diterpenes, sesterterpenes, triterpenes, and alkaloids), were submitted for biological screening to the Lilly’s Open Innovation for Phenotypic Drug Discovery (PD2-OIDD) program after successfully passing the initial online bioinformatics screen. The results for the phenotypic screening assays in the area of angiogenesis and secondary target-based biochemical kinase profiling supported by molecular docking studies for the active hits are reported along with preliminary structure-activity relationships (SAR).

## 2. Results

Seventy-one marine natural and semisynthetic product structures were sketched, converted to Molfile format and submitted to the Lilly’s PD2/OIDD online bioinformatics screen (https://openinnovation.lilly.com/dd/). These structures were subjected to the program’s bioinformatics filter system, which resulted in the exclusion of structures with insufficient novelty and/or non-druggable functionalities. This bioinformatics filter system calculates the *in silico* molecular descriptors of submitted structures including molecular weight, polar surface area, clogP, number of rotatable bonds, and number of hydrogen bond donors and acceptors. *In silico* data were used to predict the physicochemical properties that would inevitably affect pharmacokinetics, pharmacodynamics, and future druggability of submitted structures. The structures and the *in silico* molecular descriptors of accepted marine natural and semisynthetic products (38 compounds) are shown in Figures S1 and S2, respectively (Supplementary Material). Molecular weights were in the range of 248 to 593 amu, clogP of 0.75 to 8, polar surface area of 30–120 Å^2^, hydrogen bond donors (HBD) of 0 to 3 and hydrogen bond acceptors (HBA) of 2 to 8.

**Figure 1:**
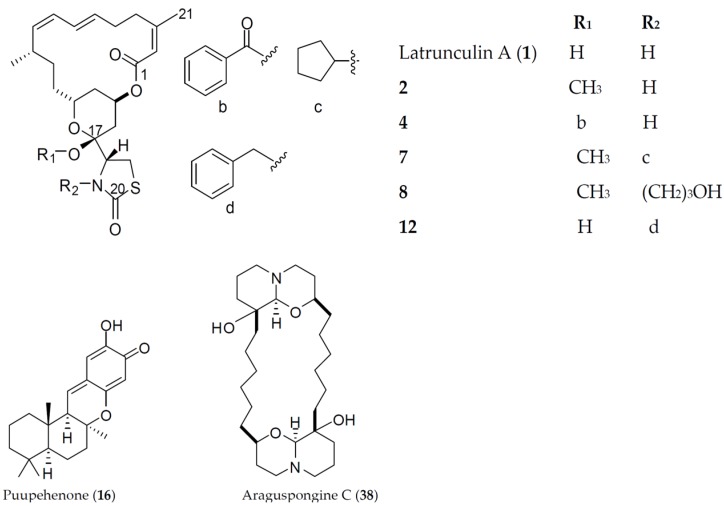
Chemical structures of the active marine natural and semisynthetic hits in Lilly's primary antiangiogenic screening assays.

Survivors from the Lilly’s bioinformatics screen were tested in angiogenesis assays utilizing the coculture of endothelial colony-forming cells (ECFCs) and human adipose-derived stem cells (ADSCs) to mimic, in part, the biological complexity of angiogenesis. The inhibition of the endothelial colony-forming cell (ECFCs) tube and nuclei areas by the active hit **1** (latrunculin A) and sunitinib (a standard antiangiogenic positive control) is shown in Figure 3S (Supplementary Material). Sunitinib displayed a dose response effect on the ECFCs’ CD31 tube area, with IC_50_ = 0.034 µM, and minimal cytotoxic effects up to 1 µM. In parallel, latrunculin A (**1**) exhibited a dose response of an active inhibitor in the ECFCs’ CD31 tube area, with an IC_50_ of 0.043 µM, while affecting nuclei area (as marker of cell toxicity) at IC_50_ almost 20-fold higher than its effective dose in ECFCs’ CD31 tube area inhibition (0.824 µM). Consequently, the related latrunculin-based natural products and semisynthetic analogs **2**–**15** (Figure 1) were further tested and exhibited potent endothelial tube formation inhibition at 10 µM in primary angiogenic screening assay. The natural product latrunculin H (**13**, the *N*-hydroxymethyl latrunculin A analog) was found to be comparable to the parent **1**, with corresponding IC_50_ value of 0.063 µM in the ECFCs’ CD31 tube area inhibition assay. However, it exhibited much more cytotoxic effect, indicated by its lower IC_50_ value (0.29 µM) in ECFCs’ CD31 nuclei area assay. The reported actin polymerization inhibitory activity of **13** was 1.35-fold more potent than that of **1**; IC_50_ values were 210 and 284 nM, respectively [8]. This may suggest different antiangiogenic mechanism/molecular target(s) for latrunculin A (**1**) rather than direct actin polymerization inhibition.

**Figure 2:**
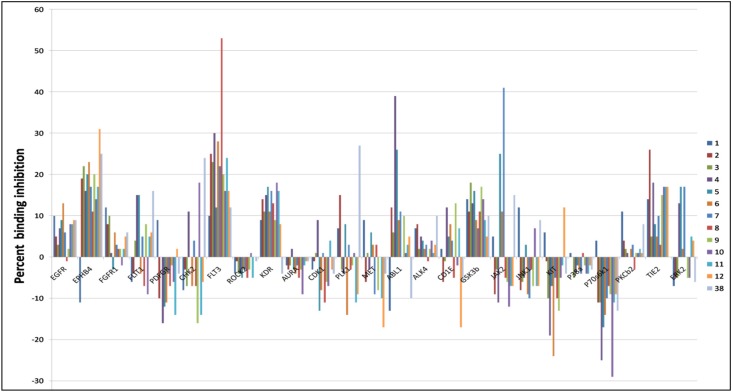
Angiogenesis kinase profiling results for bioactive natural and semisynthetic latrunculins and araguspongine C. Data represent percent of binding inhibition at 2 µM in LanthaScreen™ kinase assay. JAK2, FLT3, EphB4, ABL1, TIE2 and PLK1 were the most targeted kinases by compounds **7**, **8**, **12**, **4**, **2**, and **38**, respectively.

Because of promising antiangiogenic activities displayed by latrunculins, active hits were further screened against a wide array of kinases relevant to the angiogenesis cascade. Results indicated that latrunculin A and semisynthetic analogs displayed good selectivity toward specific kinases (Figure 2). Janus kinase 2 (JAK2), Fms-like tyrosine kinase 3 (FLT3), ephrin type-B receptor 4 (EphB4), Abelson murine leukemia viral oncogene homolog 1 (ABL1), tyrosine kinase with immunoglobulin-like and EGF-like domains 2 (TIE2), and polo-like kinase1 (PLK1) were among the most targeted kinases.

**Figure 3:**
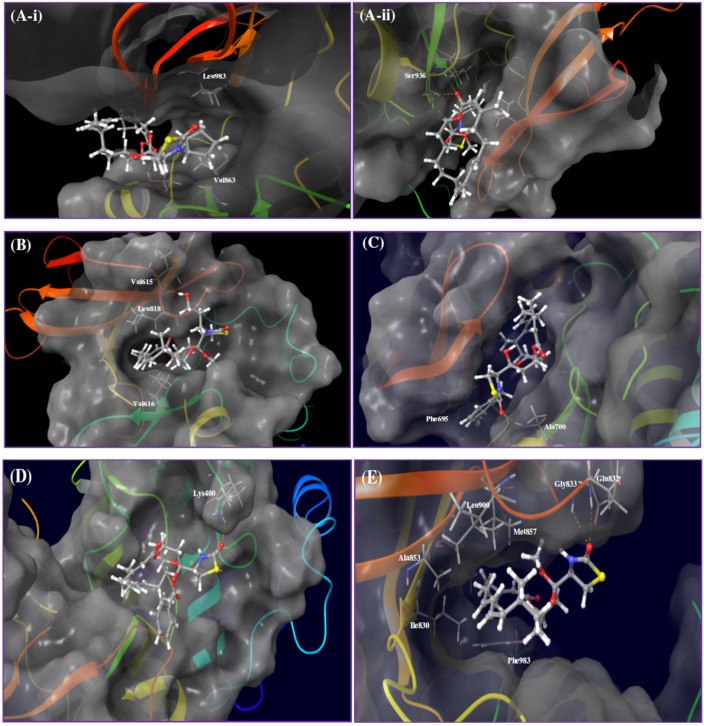
Proposed binding modes of bioactive latrunculin-based analogs in angiogenesis-targeted kinases. (**A**) Binding mode of latrunculin analog **7** in JAK2 kinase domain (PDB: 4ZIM). (**ii**) Interactions of **7** through a donor-acceptor-donor triad hydrogen bonding system (yellow dotted lines), comprising Ser936 backbone NH, lactone carbonyl oxygen of **7** and Ser936 side chain OH, respectively. (**i**) Hydrophobic contacts of the *N*-cyclopentyl ring of **7** with the side chains of Val863 and Leu983; (**B**) Binding mode of latrunculin analog **8** in FLT3 kinase domain (PDB code: 4XUF). The extended *N*-hydroxypropyl side chain of **8** forms a hydrogen bond with Val615 backbone amide carbonyl oxygen. The macrocyclic lactone is imbedded into the deep catalytic cleft and sandwiched between hydrophobic N-terminal Val616 and *C*-terminal Leu818; (**C**) Binding mode of latrunculin analog **12** in EphB4 kinase domain (PDB code: 3ZEW). Analog **12** is entrapped within the catalytic pocket and forms hydrogen bonding with Ala700 backbone amide hydrogen and *π*-*π* stacking with the hinge Phe695; (**D**) Binding mode of latrunculin analog **4** in ABL1 kinase domain (PDB code: 4WA9). The macrolide scaffold acquires in-pocket pose, while the TZD ring and the C-17-*O*-benzoyl are oriented out-of-pocket. Analog **4**’s TZD carbonyl oxygen forms a hydrogen bonding interaction with the side chain amino of the Lys400; (**E**) Binding mode of 17-*O*-methylatrunculin A (**2**) in TIE2 kinase domain (PDB: 2WQB). Analog **2**’s TZD carbonyl oxygen creates a bifurcated hydrogen bonding with backbone amide hydrogens of Glu832 and Gly833 residues. The C-17-*O*-methyl creates a hydrophobic interaction with the Val838 side chain in the *N*-terminal lobe, while the C-22-methyl is oriented to interact the with the hydrophobic side chain of the Leu830. The macrolide scaffold is buried in a hydrophobic pocket created by amino acids side chains of the *N*-terminal loop (Ile830, Ala853, Met857, Leu900, Ile902, and Phe983).

Kinase profiling results revealed that analog **7** (17-*O*-methyl-*N*-cyclopentyllatrunculin A) inhibited 41% of JAK2’s binding affinity at 2 µM. A docking study was conducted utilizing the X-ray crystal structure of the JAK2's catalytic domain to better understand how **7** interacts with JAK2. The proposed binding model revealed that **7** can reside within a constricted ATP-binding pocket, and occupy part of the main active site, nearly where the adenine base of ATP usually resides (Figure 3A-i). Surface representation of the docking model (Figure 3A-i) showed that **7** was oriented in such a way that its macrocyclic scaffold pointed towards the exit of the pocket, while the substituted fused THP and its attached TZD ring were both oriented toward the narrow and deep hydrophobic catalytic cleft (Figure 3A-i). Latrunculin analog **7** was proposed to bind the protein hinge region through donor-acceptor-donor triad hydrogen bonding system, comprising the Ser936 backbone amide hydrogen, **7**’s lactone carbonyl oxygen and Ser936’s side chain hydroxyl group, respectively (Figure 3A-ii). Moreover, analog **7**, a predominately hydrophobic molecule (clogP = 6.54), is expected to form hydrophobic contacts within the JAK2’s hydrophobic kinase pocket. The *N*-cyclopentyl moiety was sandwiched between the hydrophobic Val863 residue of the *N*-terminal lobe and the Leu983 residue of the *C*-terminal lobe (Figure 3A-i).

Latrunculin analog **8** (17-*O*-methyl-*N*-hydroxypropyllatrunculin A) inhibited 53% of the FLT3 binding affinity at 2 µM in the kinase biochemical profiling screen. Analog **8** was docked into the FLT3 kinase domain to address its possible binding mode. Results revealed that **8** can reside at the binding cleft comprising the hinge region, phosphate binding loop and the activation loop (Figure 3B). This binding domain is matching most other known small-molecule kinase inhibitors. Detailed examination of **8**’s binding mode suggested a hydrogen bonding interaction between the extended *N*-hydroxypropyl side chain at the TZD ring and the Val615’s backbone amide oxygen. The hydrophobic macrocyclic lactone scaffold was imbedded inside the deep catalytic cleft and sandwiched between the hydrophobic *N*-terminal Val616 and the *C*-terminal Leu818 residues, just prior to the activation loop (Figure 3B).

Latrunculin analog **12** (*N*-benzyllatrunculin A) inhibited 31% of the EphB4 binding affinity at 2 µM in the kinase profiling assay. Molecular docking was conducted utilizing the ATP binding pocket of EphB4 kinase domain to support *in vitro* biochemical activity. This study showed that EphB4’s bi-lobular kinase folding entrapped analog **12** within its catalytic pocket (Figure 3C). Precise examination of binding pose disclosed the tight folding of the glycine-rich loop over **12**, forcing almost a closed conformation for EphB4. Moreover, the macrocyclic lactone scaffold was directed towards the hydrophobic pocket of the kinase domain, while the C-17 free OH group and the TZD ring were both oriented out-of-pocket towards the solvent (Figure 3C). This binding mode would facilitate hydrogen-bonding interaction with the water molecules in solution state and hence stabilize the overall in-pocket binding architecture. Additionally, **12**’s binding pose enabled the TZD carbonyl oxygen to accept a hydrogen bond from the backbone amide hydrogen of the Ala700, located at the edge of the binding site within the hinge loop. Furthermore, the *N*-benzyl side chain was proposed to form *π*-*π* stacking with the nearby phenyl moiety of the Phe695, to further stabilize the overall binding conformation (Figure 3C).

Kinase screening data indicated that the ABL1 affinity binding was the most inhibited by latrunculin analog **4** (17-*O*-benzoyllatrunculin A); 39% at 2 µM. To explore how analog **4** modulates the ABL1's binding affinity, molecular docking study was pursued utilizing the X-ray crystal structure of ABL1 kinase domain. Results indicated that latrunculin **4**’s macrolide scaffold acquired in-pocket binding pose, while the TZD ring and the C-17-*O*-benzoyl were oriented out-of-pocket (Figure 3D). This conformation facilitated the creation of a hydrogen bonding between the TZD carbonyl oxygen and the side chain terminal amino of Lys400, located in the activation loop. It is obviously that the C-17-*O*-benzoyl moiety did not participate in any kind of molecular interactions with the amino acid residues within the catalytic cleft. However, the C-17-*O*-benzoyl group might help in orienting the rest of the molecule at a favorable confirmation for binding with the Lys400 at the ABL1's activation loop (Figure 3D).

Kinase profiling data indicated that latrunculin **2** (17-*O*-methyllatrunculin A) moderately reduced TIE2 binding affinity by 26% at 2 µM. To address how **2** interacts with TIE2, the prepared 3D structure of **2** was virtually docked into the catalytic pocket of TIE2's X-ray crystal structure. Results revealed that **2** can occupy the TIE2's open-conformation catalytic cleft, whereas the lipophilic macrolactone skeleton was buried in a deep hydrophobic pocket created by the side chains of several amino acids (Ile830, Ala853, Met857, Leu900, Ile902, Phe983) at the *N*-terminal loop (Figure 3E). In addition, **2** established a bifurcated hydrogen bonding between its TZD carbonyl oxygen and the backbone amide hydrogens of the Glu832 and Gly833 residues, located at the glycine-rich nucleotide-binding loop (residues 831-836). Moreover, the C-17-*O*-methyl is expected to create a hydrophobic interaction with the nearby lipophilic side chain of the Val838 in the N-terminal lobe (Figure 3E). Meanwhile, the C-22-methyl of **2** was oriented to create additional hydrophobic contact with the Ile830's side chain, in the region just prior to the glycine-rich loop.

**Figure 4:**
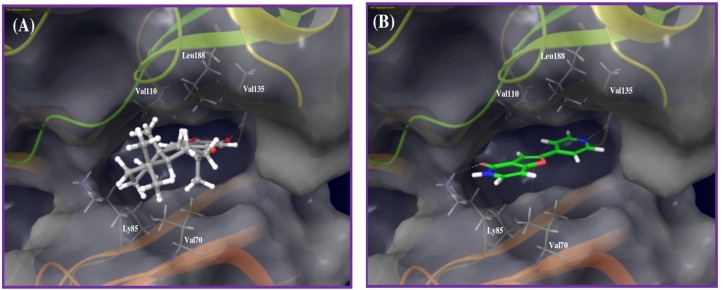
Binding modes of puupehenone and cocrystallized ligand in GSK-3β kinase domain (PDB code: 3ZRK). (**A**) Puupehenone (**16**) quinone oxygen accepts a hydrogen bond from the main chain amide hydrogen of Val135 in the hinge region. The tetracyclic hydrophobic scaffold and attached methyl groups were sandwiched between hydrophobic side chain of Val70 at the floor of the pocket, and hydrophobic side chains of Val110 and Leu188 in the ceiling of the pocket; (**B**) The cocrystallized ligand accepts hydrogen bonds from the backbone amide hydrogen of the Val135 in the hinge and side chain amino group of the Lys85 at the pocket exit.

The shikimate-derived sesquiterpene puupehenone (**16**) exhibited 107% inhibition of the endothelial tube area in Lilly OIDD’s antiangiogenic assay. Puupehenone did not progress further to the kinase profiling secondary assays. Alternatively, puupehenone was virtually screened against the available 3D structures of the PD2 kinase panel. Results suggested GSK-3β as a possible kinase target. The proposed binding mode of puupehenone in the kinase X-ray crystal structure of GSK-3β identified a critical hydrogen bonding interaction between puupehenone quinoide oxygen and main chain amide hydrogen of the Val135 of the kinase hinge (Figure 4A). In addition, the sesquiterpene skeleton was sandwiched between the hydrophobic side chains of Val70 at the floor of the pocket, and the Val110 and Leu188 in the pocket ceiling.

To validate the docking study, the X-ray cocrystallized ligand was extracted and docked into the GSK-3β catalytic pocket. Figure 4B represents the cocrystallized ligand-binding mode, where the pyridine nitrogen accepted a hydrogen bond from the backbone amide hydrogen of the Val135 in the hinge, which is the same location and interaction exhibited by puupehenone. Comparing both binding modes emphasized that the X-ray cocrystallized ligand provided an extra binding spot through a hydrogen bonding interaction with the Lys85 terminal amino group, located at the exit of the kinase pocket (Figure 4B).

**Figure 5:**
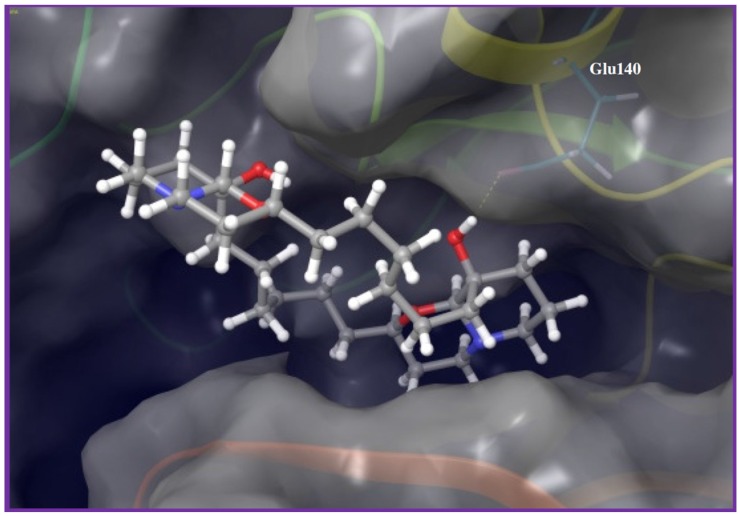
Binding mode of araguspongine C (**38**) in PLK1 kinase domain (PDB code: 3THB). Araguspongine C binds at the hinge region through a key hydrogen bond interaction between the C-9 hydroxyl group at the quinazolidine scaffold and side chain carboxylate of Glu140.

The last bioactive hit was the marine-derived macrocyclic oxaquinolizidine alkaloid araguspongine C (**38**). Assessment of **38**’s activity against the tested kinase panel revealed polo-like kinase 1 (PLK1) as the most sensitive kinase, with corresponding 27% binding inhibition at 2 µM. Based on the *in vitro* kinase profiling data, molecular modeling study was established to disclose possible molecular interactions of PLK1 and araguspongine C. Docking of **38** into the PLK1 kinase domain revealed its binding to the hinge region, through a key hydrogen bond between the tertiary alcoholic group on one of the quinazolidine ring systems and the side chain carboxylate of Glu140 (Figure 5). Moreover, the rest of the molecule was oriented out-of-pocket, exposing the other quinazolidine and the C-9 OH group towards the solvent, which may create additional hydrogen bonding with surrounding water molecules in solution state.

## 3. Discussion

Integrative collaboration between biopharmaceutical industry and academic programs strengthens drug discovery efforts and offers unique resources to translate basic biomedical research into clinical discoveries and translational developments. In this context, Eli Lilly granted academic programs access to their unique target-based and phenotypic drug discovery resources through the Phenotypic Drug Discovery Initiative (PD2/OIDD). The current study utilized this initiative via the submission of in-house marine natural product-based mini-library for phenotypic screening through Lilly’s PD2 and investigations of various assay outcomes based on medicinal chemistry perspectives. Accepted compounds were subjected to biological screening in a wide array of *in vitro* biological assays, covering the areas of diabetes/insulin secretion, osteoporosis, Alzheimer's disease, cell division and angiogenesis. Herein, we address the results of angiogenesis screening assays and relevant kinase profiling of the most active hits.

Predicted molecular descriptors of bioactive hits; calculated by Lilly’s bioinformatics system, disclosed a lower number of hydrogen bond donors (1–2), while relatively high cLogP values (4.3–6.8). Therefore, we expected hydrophobic interactions to dominate the binding of active hits with their respective molecular target(s), over the polar contacts. This hypothesis was augmented by molecular modeling and docking results. The bioactive latrunculin A (**1**) was able to inhibit ECFC tube formation with minimal effect on nuclei area at relevant doses, thereby indicating true antiangiogenic potential rather than false positive effect, mediated through cell toxicity. In addition, fine-tuning of latrunculin chemical structures was accompanied with remarkable modulation of both antiangiogenic and toxicity profiles, as in latrunculin A (**1**) and latrunculin H (**13**, the *N*-hydroxymethyl latrunculin A). This helped in shaping a preliminary structure-activity relationship (SAR), in addition to the ability to predict the binding roles for specific substituents. Utilizing molecular modeling and docking studies of the active latrunculins *versus* their respective target kinases, the hydrophobic macrolide scaffold was frequently embedded in a hydrophobic binding pocket within the kinase fold. This was in accordance with the preliminary suggestions for the contribution of hydrophobic interactions to binding. Meanwhile, the TZD *N*-substituents were varied in their binding roles among different kinases.

**Figure 6:**
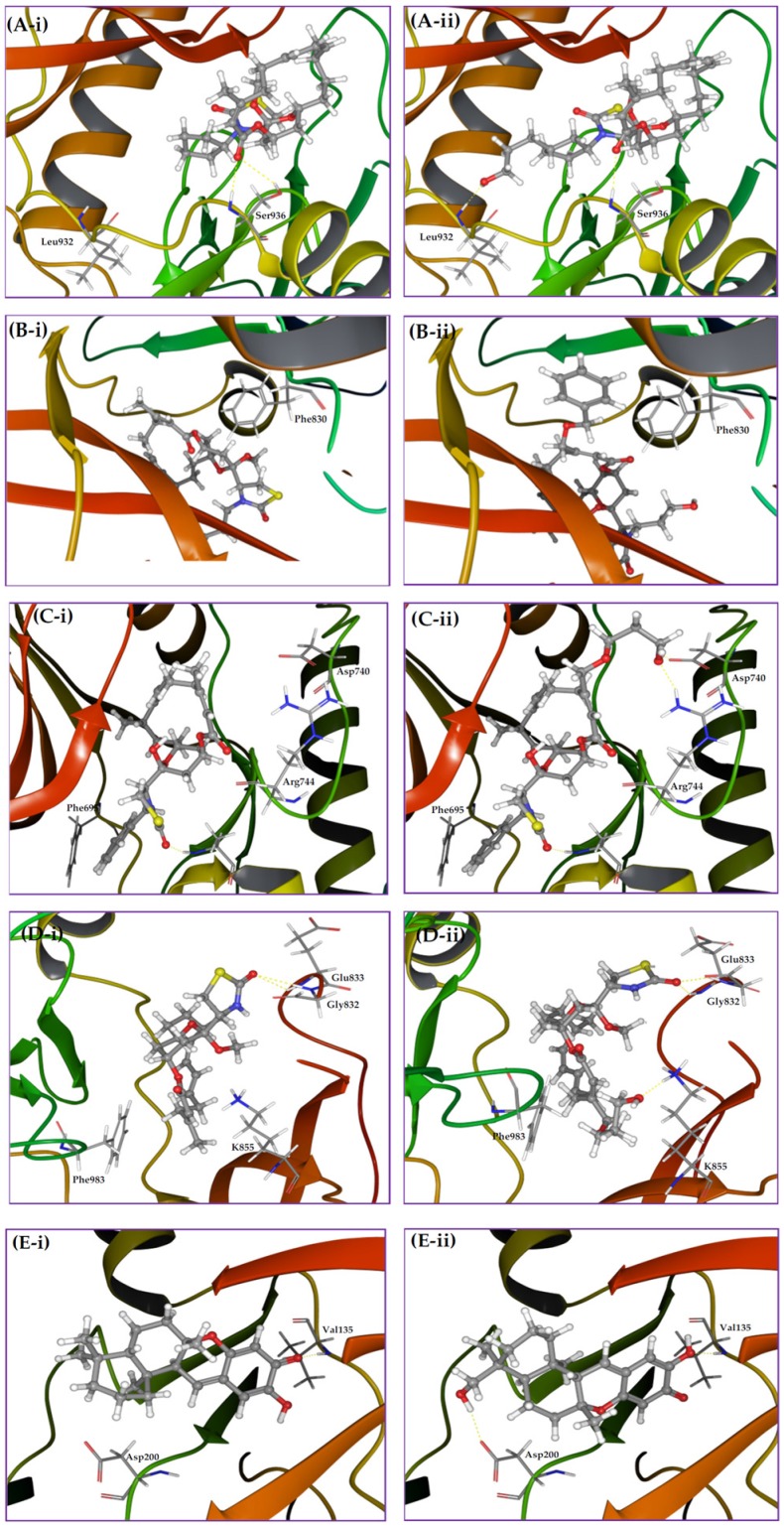
Binding modes of bioactive MNPs and their rationally designed virtual analogs in targeted kinases. (**A-i**) Binding pose of latrunculin **7** in JAK2 kinase domain showing the orientation of *N*-cyclopentyl towards a narrow pocket. (**A-ii**) Binding pose of the rationally designed analog, where the added hydroxypropyl forms a hydrogen bonding with backbone amide hydrogen of Leu932; (**B-i**) Binding pose of latrunculin **8** in FLT3 kinase domain showing the nearby phenyl side chain of Phe830. (**B-ii**) Binding pose of the rationally designed analog showing a T-stacking of the newly introduced benzyl moiety with phenyl side chain of Phe830. (**C-i**) Binding pose of latrunculin **12** in EphB4 kinase domain showing the nearby amino acids Asp740 and Arg744; (**C-ii**) Binding pose of the rationally designed virtual analog showing hydrogen bonding interactions of terminal hydroxyl of the added glycol moiety with side chain carboxylate and guanidino groups of Asp740 and Arg744, respectively (**D-i**) Binding pose of latrunculin **2** in TIE2 kinase cleft showing the C-21 methyl on *β*-carbon of the *α-β* unsaturated macrolactone buried in a shallow sub-pocket; (**D-ii**) Binding pose of the rationally designed virtual analog showing a hydrogen bonding interaction of the terminal hydroxyl group of the newly attached 1,4-butadiol with side chain amino of Lys855. Meanwhile, the 4C carbon linker forms a hydrophobic interaction with aromatic side chain of Phe983; (**E-i**) Binding pose of puupehenone in GSK-3β kinase domain showing Asp200 in a close vicinity to the decalin moiety; (**E-ii**) Binding pose of a virtual analog showing a hydrogen bonding interaction of newly introduced hydroxyl group with side chain carboxylate of Asp200.

Exploration of the chemical environment surrounding each bioactive MNP within its corresponding kinase pocket is a potential strategy to rationally design new analogs with enhanced binding characteristics. Therefore, multiple molecular modifications were proposed at specific spots to probe additional binding interactions. The *N*-cyclopentyl of the latrunculin analog **7** was oriented in a relatively narrow pocket within JAK2 kinase domain, which comprises an α-helix and a part of an extended loop (Figure 6A-i). Virtual analogs with different substituents on the cyclopentyl ring were proposed and docked into JAK2’s kinase cleft. The hydroxypropyl represented an optimal substituent for interacting with the backbone amide hydrogen of Leu932 at the floor of the pocket, along with maintaining the original binding pose (Figure 6A-ii). This inevitably will improve JAK2 binding affinity and thus the antiangiogenic activity. The phenyl side chain of Phe830 in FLT3 kinase pocket was oriented in a close proximity to the C-21 methyl on the β-carbon of the α,β-unsaturated macrolactone of the latrunculin analog **8** (Figure 6B-i). Allylic bromination of this methyl group followed by electrophilic substitution with benzyl alcohol should introduce an aromatic moiety appropriate for π-π stacking with the Phe830 side chain. Subsequent docking of this virtual analog disclosed an effective T-stacking of the introduced benzyl moiety with the targeted Phe830 phenyl side chain (Figure 6B-ii). Moreover, extension of latrunculin **8**’s TZD *N*-substituent length with hydrophilic moieties can probably enhance FLT3 binding potency and increase possibility of hydrogen bonding formation with the surrounding water molecules in the solution state, since *N*-substituent is oriented out-of-pocket towards the solvent molecules (Figure 3B).

In a similar tactic, the binding pose of latrunculin analog **12** in EphB4 receptor showed that the C-21 methyl group resided in a close proximity to the polar side chains of amino acids Asp740 and Arg744 (Figure 6C-i). Thus, molecular extension of latrunculin analog **12** was devised with polar substituents to probe additional hydrogen bonding interactions. Allylic bromination of the C-21 methyl followed by electrophilic substitution with propylene glycol would introduce a short polar side extension at this position. Subsequent docking of this new virtual analog disclosed two new hydrogen bonds between the free hydroxyl group of the glycol moiety and the side chains carboxylate and guanidino groups of Asp740 and Arg744, respectively (Figure 6C-ii). In addition, latrunculin analog **12**’s *N*-substituents bearing aromatic moieties with zero or one carbon linker to the TZD ring will be optimal for the EphB4 binding because longer linkers may create steric clashes or even deviate the aromatic ring away from the optimal position for appropriate *π*-*π* stacking (Figure 3C). This was further supported with the lower EphB4 binding affinity inhibition with latrunculin analog **3** (18% at 2 µM), which bears an *N*-phenylethyl moiety, representing a two-carbon linker example. In contrast, the non-substituted TZD ring was proposed to be preferable for ABL1 inhibition by latrunculin analog **4** because the *N*-substituents (especially bulky ones) would create steric clashes and thus might disrupt the overall binding conformation (Figure 3D). This hypothesis was further augmented by the reduced ABL1 binding inhibition potencies of latrunculins bearing *N*-substituents including: *N*-phenethyl (3), *N*-ethyl (**6**), and *N*-cyclopentyl (**7**), to 1%, 9%, and 11% at 2 µM, respectively.

The docking pose of latrunculin analog **2** in TIE2 kinase cleft revealed that the C-21 methyl group is buried in a shallow sub-pocket at the core of the kinase domain (Figure 6D-i). Two sub-pocket amino acids were targeted for additional interactions; K855 and Phe983. A proposed allylic bromination of the C-21 methyl followed by electrophilic substitution with alkene 1,ω*-*diols should afford these proposed polar analogs. Docking of these virtual analogs, with different carbon atoms connecting diol groups, into the TIE2 kinase domain showed interesting results. Four-carbon linker (using butane 1,4-diol with the allyl bromide intermediate) was found to be an optimal spacer to reach the targeted amino acids in the targeted sub-pocket with improved binding affinity. Thus, the terminal hydroxyl group was able to create a hydrogen bonding with side chain amino group of Lys855 (Figure 6D-ii). In addition, a hydrophobic interaction of the four-carbon linker with the phenyl side chain of Phe983 was also observed. Collectively, these additional binding interactions will inevitably potentiate latrunculin analog **2**-mediated TIE2 kinase inhibition.

Marine sesquiterpene quinones constitute a prominent class of natural products previously reported to have antiangiogenic potentials [12]. However, no previous data reported address the binding mode of this unique scaffold at possible angiogenesis-targeted kinases. Molecular docking of puupehenone (**16**) into GSK-3β kinase domain suggested a binding mode similar to the X-ray cocrystallized ligand. Comparing both binding modes emphasized an extra binding spot provided by the cocrystallized ligand, through a hydrogen bonding interaction with Lys85 terminal amino group, which is located at the exit of the pocket (Figure 4B). Moreover, **16** was moderately directed out-of-pocket, probably to avoid unfavorable clash with the same side chain amino group of Lys85. Therefore, hydroxylation of **16**’s terminal *trans*-decalin can provide additional epitopes to interact with polar side chains of the amino acids at the entrance of the pocket and thus enhance the binding potency. This can be accomplished through selective chemical or microbial hydroxylation. To test this hypothesis, multiple virtual analogs with different hydroxylation patterns on the *trans*-decalin were proposed to probe additional polar interactions, especially with the nearby Asp200 (Figure 6E-i). The virtual analog with puupehenone’s C-4 α-methyl oxidized to a hydroxymethyl, maintained the original interaction with the critical Val135, in addition to a new hydrogen bonding interaction with the side chain carboxylate of the targeted Asp200, with flipped main scaffold. This kind of structural modification can be an excellent starting point to design future new puupehenone-based GSK-3β inhibitors.

The last bioactive hit, the marine alkaloid araguspongine C (**38**), was discovered to target PLK1 in the Lilly’s kinase profiling module. PLK1 is implicated in the regulation of mitotic machinery and is overexpressed in many cancer cells [44,45,46]. In addition, PLK1 was found to interact with the tumor suppressor hamartin and thus positively regulates the mechanistic target of rapamycin complex 1 (mTORC1), which in turn negatively regulates the autophagy [47]. A recent study by Valianou and colleagues described the apoptosis induction and autophagy attenuation in response to PLK1 inhibition [48]. However, these pharmacological effects were specific to hamartin-deficient cells, with little effect of hamartin re-expressing cells. Meanwhile, autophagy induction in HER2-estrogen-dependent breast cancer cells by araguspongine C was through Met/HER2 dual inhibition [13]. Indeed, this effect was specific only to the HER2-overexpressing BT-474 breast cancer cells. Thus, both Valianou *et al.* [48] and Akl *et al.* [14] results were cell-specific and the finding of araguspongine C’s ability to inhibit PLK1 in cell-free assay is novel and can warrant future studies.

In conclusion, the biological space of bioactive MNPs is still not fully explored. Initiatives offered by biopharmaceutical industry provided unique resources to several academic groups to advance innovative biomedical research and drug discoveries. This sort of partnership is likely to result in more discoveries of novel, selective and potent future drug candidates and benefit the medical community. Selective phenotypic assays and computer-aided drug discovery technologies should be in harmony to discover unique bioactive scaffolds. Rational design of MNPs-based analogs to probe additional binding interactions with angiogenesis-related kinases is a key step to prepare superior bioactive analogs. Collectively, MNPs can be the future parents of next-generation antiangiogenic drugs.

## 4. Materials and Methods

### 4.1. General Experimental Procedures

Purity and stability of test compounds were checked by spectroscopic techniques. ^1^H and ^13^C NMR spectra were recorded at 400 and 100 MHz, respectively, in appropriate deuterated NMR solvents, using TMS as an internal standard, on a JEOL Eclipse ECS-400 NMR spectrometer (Peabody, MA, USA). The ESI-MS experiments were conducted using a 3200 Q-trap LC/MS/MS system (Applied Biosystems, Foster City, CA, USA) using Analyst version 1.4.1 software (MDS SCIEX, Foster City, CA, USA).

### 4.2. Test Compounds

All pure marine natural and semisynthetic analogs, >95% purity by HPLC and/or NMR analyses, were packed in barcoded amber glass vials under N_2_ and overnight-shipped to Lilly’s OIDD program.

Latrunculins were isolated from the Red Sea sponge *Negombata magnifica* [7]. Araguspongine C was isolated from the Red Sea sponge *Xestospongia exigua* [49]. Puupehenone was isolated from the Hawaiian sponge *Hyrtios* sp. [50]. Compounds were purified by open column chromatography either using Sephadex LH-20 and/or Si gel 60 as stationary phases and their identity and purity were confirmed by comparison with literature spectroscopic data.

### 4.3. Biological Experiments

#### 4.3.1. Cell Lines

Human endothelial colony-forming cells (ECFC, Engenator) were cultured in Endothelial Growth Media-2 Microvascular (EGM-2MV, Lonza Ltd., Basel, Switzerland) media containing 10% FBS (EGM-2MV + FBS). ECFCs were expanded to passage 7, rapidly frozen and stored over liquid N_2_ until use [51]. Human adipose-derived stem cells (ADSC, ZenBio, Durham, NC, USA) were derived from pooled donor samples, cultured in EGM-2MV + FBS, expanded to passage 5, rapidly frozen and stored over liquid N_2_ until used.

#### 4.3.2. Endothelial Colony-Forming/Adipose-Derived Stem Cells (ECFC-ADSC) Coculture

ECFCs were quickly thawed, diluted into pre-warmed EGM-2 media containing 10% FBS (EGM-2 + FBS), and incubated for 24 h at 37 °C, in a humidified incubator at 5% CO_2_ [51]. ECFCs were washed with PBS, suspended with trypsin, and expanded EGM-2 + FBS for 48 h at 37 °C, in a humidified incubator at 5% CO_2_. ADSCs were quickly thawed and diluted into pre-warmed EGM-2MV + FBS. The ADSCs were collected by centrifugation and suspended in Optimized Media (TCS Cellwork). ADSCs (5000/well) were added to a 384-well Cell Bind plate (Corning, Manassas, VA, USA), incubated at rt for 5 min, and then placed in a 5% CO_2_ incubator overnight at 37 °C. Cultured ECFC were washed with PBS, suspended with trypsin and diluted in EGM2 + FBS. Cells were collected by centrifugation, suspended in Optimized Media and passed through a sterile 23-gauge needle. ECFCs (500/well) were overlaid onto the ADSC feeder layer and returned to the 5% CO_2_ incubator. After 2 h incubation, rhVEGF (10 ng/mL) and test compounds at the indicated concentrations were added (0.5% (*v*/*v*) DMSO final) and incubated for 96 h at 37 °C in a 5% CO_2_ incubator. Maximum and minimum responses correspond to rhVEGF + 0.5% (*v/v*) DMSO or rhVEGF + 500 nM sunitinib (Sutent^®^, Pfizer, ≥98%), respectively [51].

#### 4.3.3. Endothelial and Nuclear Staining

ECFC-ADSC cocultures were fixed with 3.7% HCHO in PBS for 20 min, treated with 0.1% TX-100-PBS for 20 min, washed twice with PBS, and then incubated with 63 ng/mL mouse Anti-Human CD31 antibody (BD Pharmigen, Franklin Lakes, NJ, USA) in PBS containing 1% BSA at 4 °C overnight. Following PBS wash, samples were then incubated with 3 μg/mL Alexa 488 goat secondary antibody in PBS for 1 h and then washed twice with PBS before staining cellular DNA with Hoechst (2 μg/mL) [51].

#### 4.3.4. Cellular Imaging

Fixed and stained ECFC-ADSC cocultures were analyzed with an Array Scan VTI (Thermo Scientific, Madison, WI, USA) using a 5× objective and collecting four non-adjacent frames per well; nuclei and CD31 were visualized using the XF93-Hoechst and XF93-Alexa-488 dichroic mirror emission filter pairs, respectively. Endothelial tube features were determined with the Cellomics Tube Formation V3 application by analysis of the CD31 channel. Relative cell death in the ECFC-ADSC coculture was measured by the loss of valid nuclear objects as determined by the Target Activation V3 application [51].

### 4.4. Biochemical Kinase Profiling

LanthaScreen™ Kinase Assay (Life Technologies, Grand Island, NY, USA) was used with a time-resolved fluorescence resonance energy transfer (TR-FRET) format to assess kinases binding inhibitory effects of test compounds [52].

#### 4.4.1. Molecular Modeling

The *in silico* experiments were carried out using Schrödinger molecular modeling software package installed on an iMac 27-inch Z0PG workstation with a 3.5 GHz Quad-core Intel Core i7, Turbo Boost up to 3.9 GHz, processor and 16 GB RAM (Apple, Cupertino, CA, USA).

#### 4.4.2. Protein Structure Preparation

The X-ray crystal structures of the human kinases JAK2 (PDB code: 4ZIM) [53], FLT3 (PDB code: 4XUF) [54], EphB (PDB code: 3ZEW [55], ABL1 (PDB code: 4WA9) [56], TIE2 (PDB code: 2WQB) [57], GSK-3β (PDB code: 3ZRK) [58], and PLK1 (PDB code: 3THB) [59], were retrieved from the Protein Data Bank [60]. The kinase domain for each protein was prepared using the Protein Preparation Wizard of the Schrödinger molecular modeling suite [61]. Protein structures were reprocessed by assigning bond orders, adding hydrogens, creating disulfide bonds and optimizing H-bonding networks using PROPKA (Jensen Research Group, Copenhagen, Denmark). Finally, energy minimization with a root mean square deviation (RMSD) value of 0.30 Å was applied using an Optimized Potentials for Liquid Simulation (OPLS_2005, Schrödinger, New York, NY, USA) force field.

#### 4.4.3. Ligand Structure Preparation

The structures of all compounds used in the docking studies were 2D sketched using the Maestro 9.3 panel (Maestro, version 9.3, 2012, Schrödinger, New York, NY, USA). The LigPrep 2.3 module (LigPrep, version 2.3, 2012, Schrödinger, New York, NY, USA) of the Schrödinger suite was utilized to generate the 3D structures and to search different conformers. The Optimized Potentials for Liquid Simulation (OPLS_2005, Schrödinger, New York, NY, USA) force field was applied to geometrically optimize the ligands and compute partial atomic charges. Finally, at most, 32 poses per ligand were generated with different steric features for the subsequent docking studies.

#### 4.4.4. Molecular Docking

The prepared X-ray crystal structures of kinase domains were employed to generate receptor energy grids using the default value of the protein atomic scale (1.0 Å) within the cubic box centered on the cocrystallized ligands. Following the receptor grids’ generation, each prepared ligand was docked into the specified kinase, using the Glide 5.8 module (Glide, version 5.8, 2012, Schrödinger, New York, NY, USA) [62].

### 4.5.Statistical Validation of the Assays

Lilly’s uses the *Z*’ score metric for interplate and interexperimental variation of signal window to ensure operation robustness [63,64]. The *Z*’ value over multiple days was 0.42 for angiogenesis-related assays [51]. The reproducibility of potency determination was evaluated by experimentally determining the assay minimum significant ratio (MSR); if two compounds have a within-run potency ratio >MSR, then the potency difference between the compounds are statistically significant [65]. The MSR value was 2.2 for angiogenesis-related assays [51].

## Supplementary Materials

The following are available online at www.mdpi.com/1660-3397/14/3/57/s1, Figure S1: Chemical structure of marine natural products library members accepted in Eli Lilly angiogenic screening assays, Figure S2: *In silico* molecular descriptors of compounds **1**–**38** calculated by Eli Lilly’s bioinformatics system. (**a**) Molecular weight. (**b**) cLogP. (**c**) Polar surface area. (**d**) Number of H-bond donors. (**e**) Number of H-bond acceptors. (f) Number of rotatable bonds, Figure S3: Effects of latrunculin A and sunitinib on the endothelial colony-forming cells (ECFCs) in angiogenesis assays. (**a**) Concentration response curve of latrunculin A (**i**) and sunitinib (**ii**) treatments on endothelial colony-forming cells’ (ECFCs) CD31 tube area. (**b**) Concentration response curve of latrunculin A (**i**) and sunitinib (**ii**) treatments on endothelial colony-forming cells’ (ECFCs) CD31 nuclei area.

## Abbreviations

MNPs	Marine natural products
FDA	United States Food and Drug Administration
EMA	European Medicines Agency
OIDD	The Lilly Open Innovation Drug Discovery Program
TZD	Thiazolidinon-2-yl
THP	Tetrahydropyran
JAK	Janus kinase
STATs	Signal transducers and activators of transcription
VEGF	Vascular endothelial growth factor
FLT3	Fms-like tyrosine kinase 3
RTK	Receptor tyrosine kinase
RAS	Rat sarcoma viral oncogene homolog
PI3K	Phosphatidylinositol-4,5-biphopshate 3-kinase
Ephrin	Eph receptor interacting protein
ABL1	Abelson murine leukemia viral oncogene homolog 1
CML	Chronic myeloid leukemia
bFGF	Basic fibroblast growth factor
TIE	Tyrosine kinases with immunoglobulin-like and EGF-like domains
VEGFRs	Vascular endothelial growth factor receptors
Grb2	Growth factor receptor-bound protein 2
EGF	Epidermal growth factor
Ang	Angiopoietin
GSK3-β	Glycogen synthase kinase-3-beta
PLK	Polo-like kinase
SAR	Structure-activity relationship
ECFCs	Endothelial colony-forming cells
ADSCs	Human adipose-derived stem cells
cLogP	Calculated logarithm of partition coefficient
HBD	Hydrogen bond donor
HBA	Hydrogen bond acceptor
