# Supplementary material for: Discovery of Novel Antiangiogenic Marine Natural Product Scaffolds
Source: Mar Drugs. 2016 Mar 11;14(3):57. doi: 10.3390/md14030057 (PMC4820311; doi:10.3390/md14030057)
Supplement: Supplementary file 1 [file marinedrugs-14-00057-s001.pdf]

Supplementary Materials: Discovery of Novel Antiangiogenic Marine Natural Product Scaffolds

Hassan Y. Ebrahim and Khalid A. El Sayed *

Macrolides

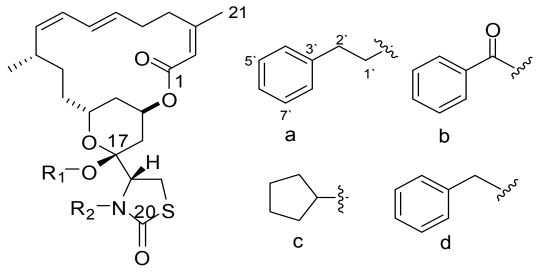
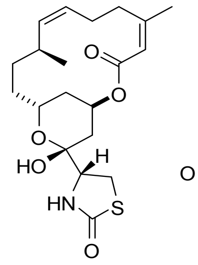
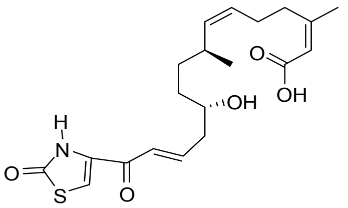

	R_1	R_2
Latrunculin A (**1**)	H	H
**2**	CH_3	H
**3**	a	H
**4**	b	H
**5**	CH_3	CH_3
**6**	CH_3	C_2H_5
**7**	CH_3	c
**8**	CH_3	$(CH_2)_3OH$
**9**	CH_3	d
**10**	CH_3	b
**11**	H	C_2H_5
**12**	H	d
Latrunculin H (**13**)	H	CH_2OH

Latrunculin B (**14**) Latrunculin T (**15**)

Sesquiterpenes

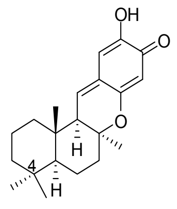
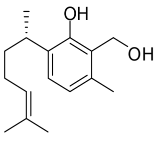
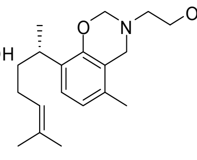
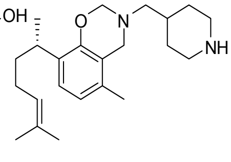
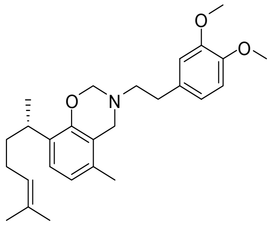

Puupehenone (**16**) Curcuphenols: **17** **18** **19** **20**

Diterpenes

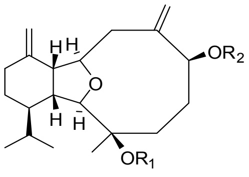

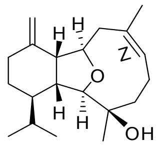

(6*Z*)-Cladiellin (**25**)

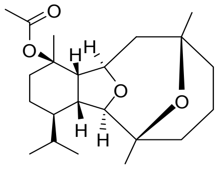

Polyanthelin A (**26**)

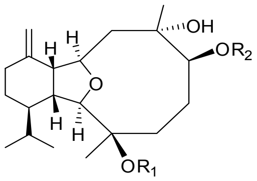

	R_1	R_2
Cladiellisin (**21**)	H	H
3-Acetyl cladiellisin (**22**)	Ac	H
3,6-Diacetyl cladiellisin (**23**)	Ac	Ac
Cladiellisin-6-butylcarbamate (**24**)	H	$CONHC_4H_9$

	R_1	R_2
Sclerophytin A (**27**)	H	H
Sclerophytin F methylether (**28**)	H	CH_3
Sclerophytin B (**29**)	Ac	H

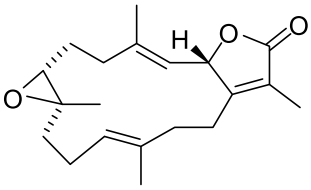

Sarcophine (**30**)

Sesterterpenes

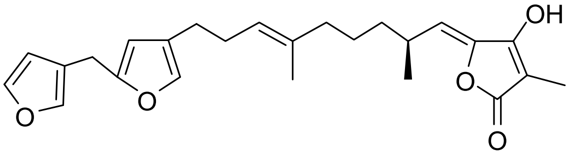

Ircinin-1 (**31**)

Triterpenes

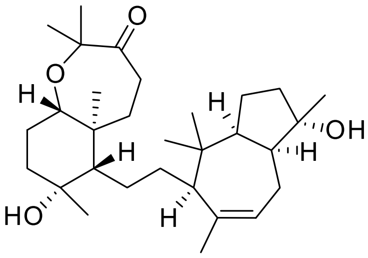

Sipholenone A (**32**)

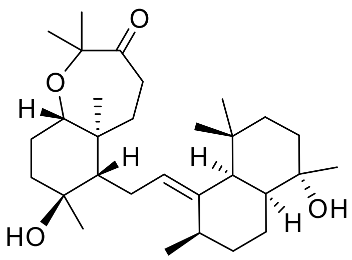

Sipholenone E (**33**)

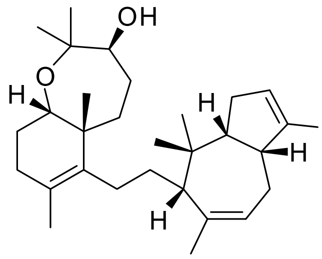

Anhydrosipholenol A (**34**)

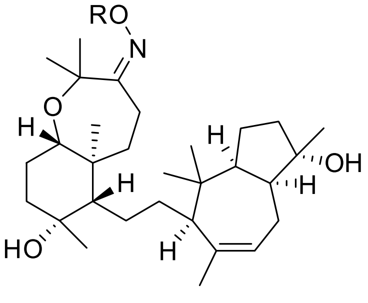

	R
Sipholenone A-4*Z*- *N*-benzoyloxime (**35**)	Benzoyl
Sipholenone A-4*Z*-*N*-benzyloxime (**36**)	Benzyl

<u>Alkaloids</u>

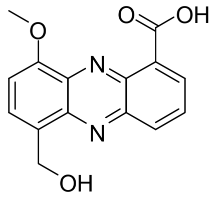

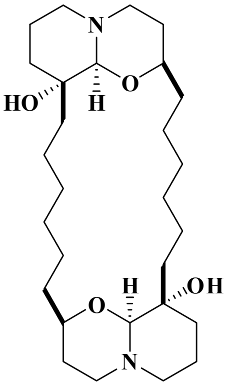

Griseoluteic acid (**37**) Araguspongine C (**38**)

Figure S1. Chemical structures of marine natural products (MNPs) library members accepted in Eli Lilly angiogenic screening assays.

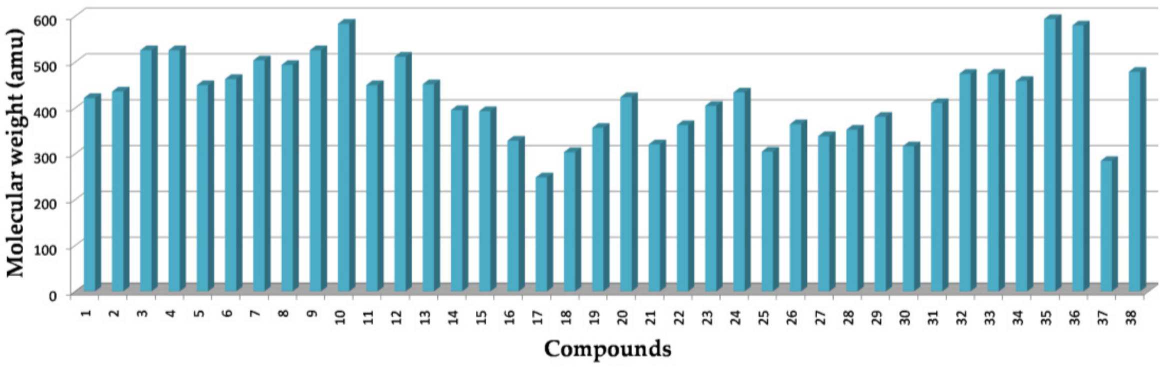

(**a**)

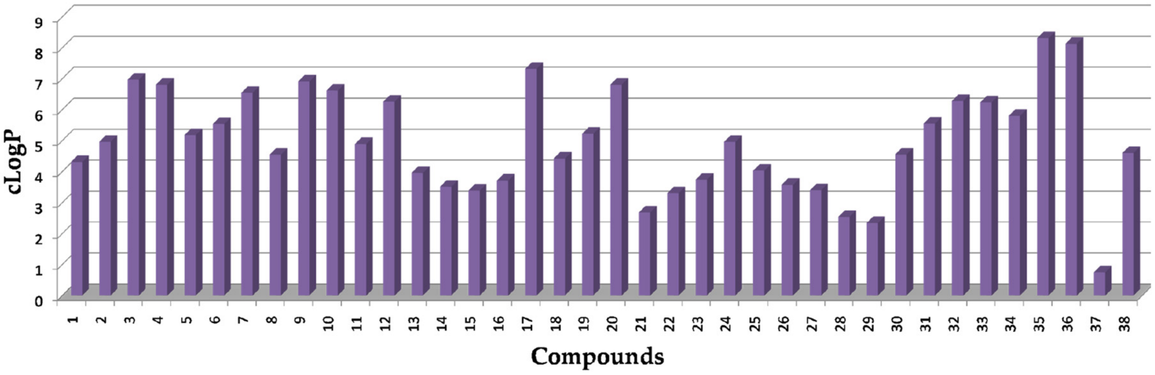

(**b**)

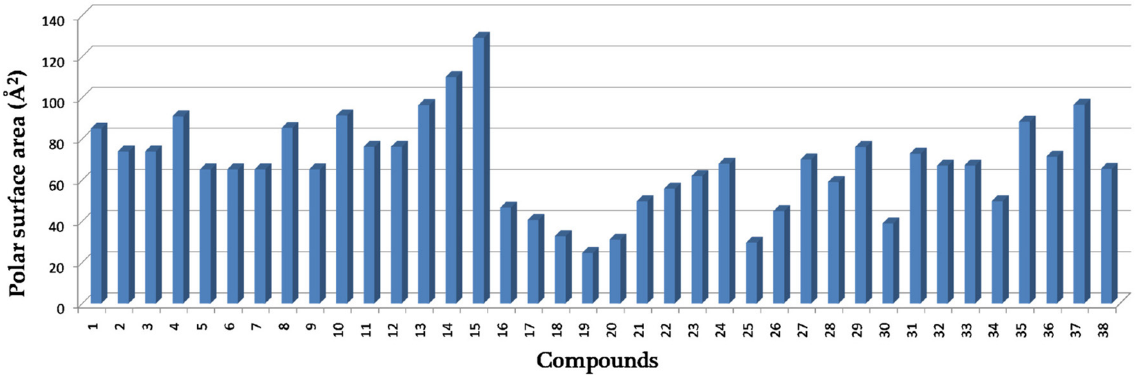

(**c**)

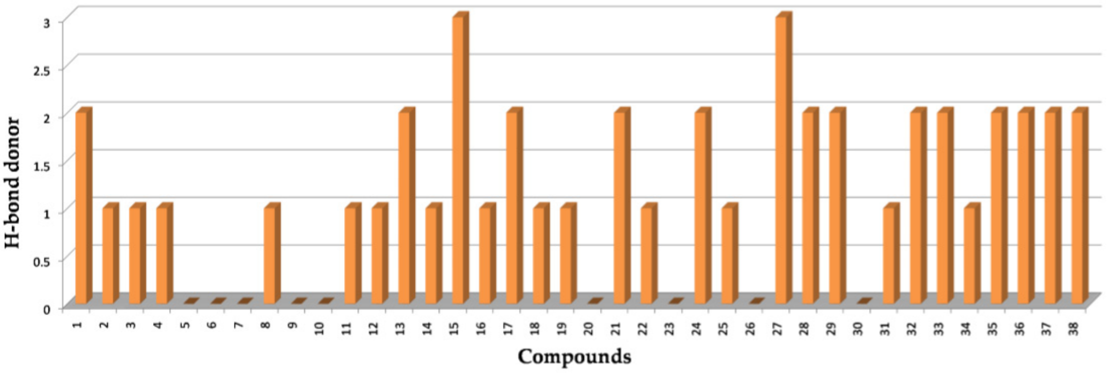

(**d**)

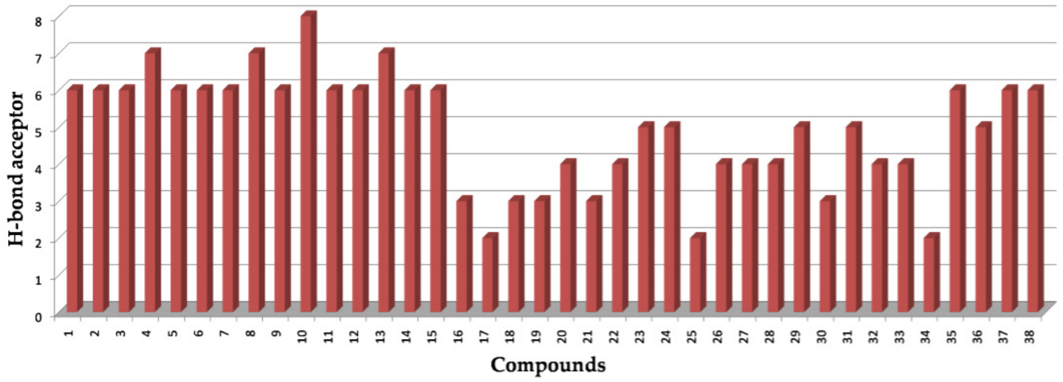

(**e**)

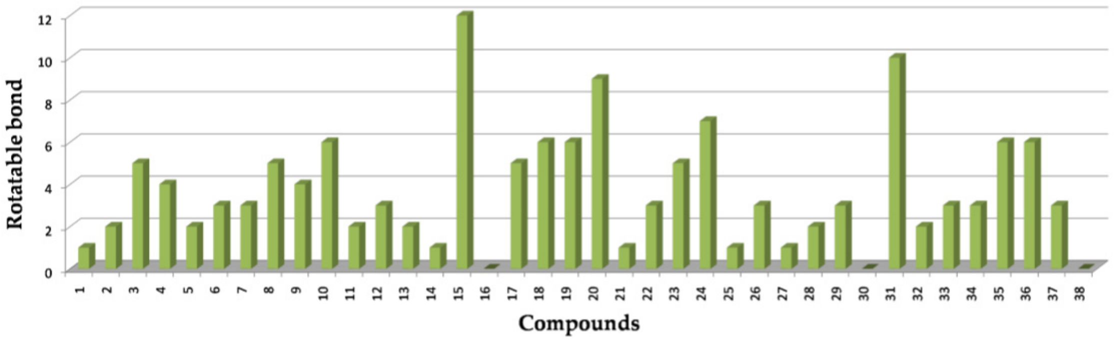

(**f**)

Figure S2. *In silico* molecular descriptors of compounds **1-38** calculated by Eli Lilly's bioinformatics system. (**a**) Molecular weight. (**b**) cLogP. (**c**) Polar surface area. (**d**) Number of H-bond donors. (**e**) Number of H-bond acceptors. (**f**) Number of rotatable bonds.

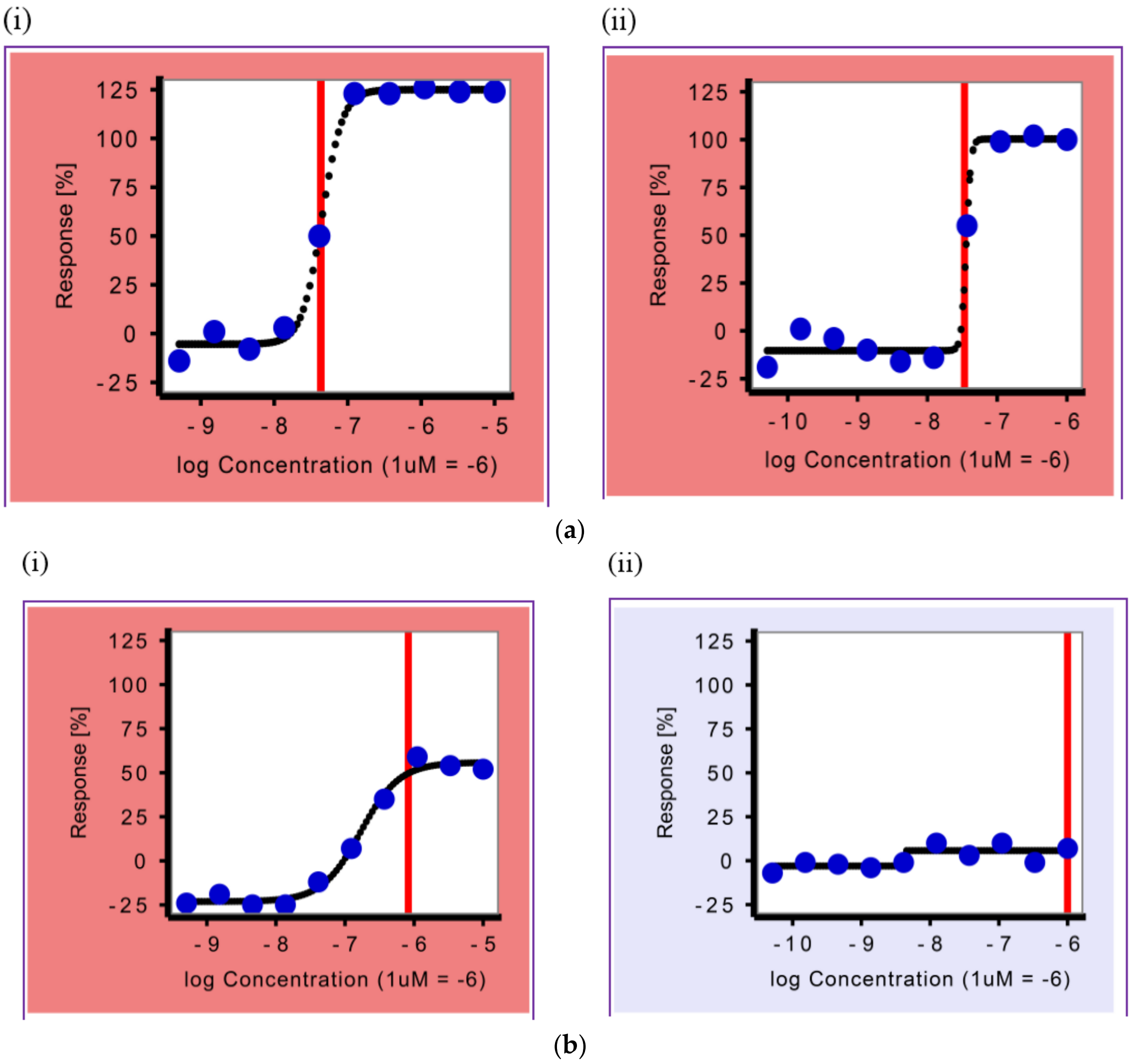

Figure S3. Effects of latrunculin A (**1**) and sunitinib on the endothelial colony forming cells (ECFCs) in angiogenesis assays. (**a**) Concentration response curve of latrunculin A (**i**) and sunitinib (**ii**) treatments on endothelial colony forming cells (ECFCs) CD31 tube area. (**b**) Concentration response curve of latrunculin A (**i**) and sunitinib (**ii**) treatments on endothelial colony forming cells (ECFCs) CD31 nuclei area.
